# CDC foundation supports emerging infectious disease projects.

**DOI:** 10.3201/eid0202.960222

**Published:** 1996

**Authors:** C Stokes


### BSE Meeting at CDC

The recent report of a new variant of Creutzfeldt-Jakob disease (V-CJD) in Great Britain and the possible link between the disease and bovine spongiform encephalopathy (BSE) has raised a number of health and safety concerns (1,2). On April 8, 1996, CDC organized a meeting of U.S. agency representatives to review information about the report of U.K. cases and about efforts to identify the existence of BSE and V-CJD in the United States. The meeting covered the scientific evidence for the report of V-CJD; recommendations from a meeting of international experts organized by the World Health Organization on April 2-3; and the current and proposed activities of U.S. agencies with regard to BSE and V-CJD.

Among the observations made during the meeting were the following:

- There is no evidence from U.S. surveillance activities or from scientific studies to indicate that BSE exists in the United States.
- Active surveillance for BSE is conducted by the U.S. Department of Agriculture (USDA). All cattle presented for slaughter in the United States are observed for signs of central nervous system (CNS) disorders. Livestock showing CNS signs are condemned and not allowed to enter the slaughter plant or to become part of the human food supply. Since 1990, laboratory testing of nearly 2,800 brain specimens from cattle with CNS signs has shown no evidence of BSE.
- No U.K. cattle or ruminant-based feed has been imported into the United States since July 1989, when USDA banned the importation of cattle and cattle products from the United Kingdom. U.K. cattle imported before the ban will be destroyed as a precaution to ensure that these animals do not enter the food chain (human or animal).
- The Food and Drug Administration plans to issue a ban on ruminant-to-ruminant feeding in the United States.
- Additional research is needed on the characterization of the causative agent of BSE and on the epidemiology, rapid laboratory diagnosis, and pathogenesis of BSE and CJD.
- The Centers for Disease Control and Prevention (CDC) monitors the occurrence of CJD in the United States through surveillance and special epidemiologic studies (3). On the basis of mortality surveillance from 1979 to 1993, the annual incidence of CJD remained stable at approximately one case per million persons. In the United Kingdom, five of eight patients who died of V-CJD since May 1995 were younger than 30 years of age; by comparison, in the United States, CJD deaths among persons younger than 30 years are extremely rare (fewer than 5 per billion per year). CDC's efforts will be expanded to include active surveillance studies at four Emerging Infections Program sites (Connecticut, Minnesota, Oregon, and the San Francisco area) and in Atlanta to provide more up-to-date information on the occurrence of CJD and to verify the absence or presence of V-CJD.

Future cooperative efforts among U.S. agencies, industry, and other interested parties in response to the report of V-CJD are planned. The report of the April 8 meeting at CDC can be accessed on the CDC NCID Web site (connect to http://www.cdc. gov/ncidod/ncid.htm; the report is under New, Reemerging, and Drug-Resistant Infections).

### References

1. Will RG, Ironside JW, Zeibler M, et al. A new variant of Creutzfeldt-Jakob disease in the UK. Lancet 1996;347:921-5.
2. CDC. World Health Organization consultation on public health issues related to bovine spongiform encephalopathy and the emergence of a new variant of Creutzfeldt-Jakob disease.MMWR 1996;45:295-6, 303.
3. Holman RC, Khan AS, Kent J, Strine TW, Schonberger LB. Epidemiology of Creutzfeldt-Jakob disease in the United States, 1979-1990: analysis of national mortality data. Neuroepidemiology 1995;14:174-81.

### CDC Foundation Supports Emerging Infectious Disease Projects

The National Foundation for the Centers for Disease Control and Prevention, Inc. (NFCDC), a not-for-profit corporation established by Congress to support CDC's mission, announced in August 1995 that one of its initial funding efforts would be in the area of antibiotic-resistant diseases.

Also in the area of infectious diseases, NFCDC has recently received a gift from a Pennsylvania foundation to establish fellowships for two representatives from nongovernmental organizations to spend 1 month at CDC learning about the agency's AIDS/HIV support services and activities and advising CDC on the type of support nongovernmental agencies need in this area.

The foundation creates new and enhanced financial and program partnerships between CDC and American and international businesses and industries, philanthropic organizations, foreign governments, international organizations, and concerned individuals. An initial grant of $1 million from the Robert W. Woodruff Foundation was awarded to help establish NFCDC in Atlanta.

The NFCDC board of directors plans to first fund projects that reflect the following themes: 1) strengthening global health capacity; 2) promoting healthy lifestyle choices with initial emphasis on adolescent health; 3) improving the quality and communication of public health information, with initial emphasis on development of information technology and networks; and 4) promoting prevention in health care delivery systems, in particular, managed-care systems.

According to foundation executive director Charlie Stokes, tax-deductible gifts to NFCDC represent an investment in CDC's efforts to address current or emerging health problems that threaten U.S. citizens and citizens of the world. Gifts may be designated for a specific purpose or be given without restriction to be used for CDC's greatest needs. Federal employees can make gifts to the foundation through the annual Combined Federal Campaign.

For further information about NFCDC, contact

**Charlie Stokes, Executive Director**
NFCDC
The Hurt Building
50 Hurt Plaza, Suite 765
Atlanta, GA 30305
phone: 404-653-0790; fax: 404-653-0330
e-mail: seg4@nfcdc.em.cdc.gov

